# Modification of thermal and electrical characteristics of hybrid polymer nanocomposites through gamma irradiation for advanced applications

**DOI:** 10.1186/s11671-024-03972-3

**Published:** 2024-02-22

**Authors:** C. M. Kavitha, K. M. Eshwarappa, Shivakumar Jagadish Shetty, S. C. Gurumurthy, Srivathsava Surabhi, T. Niranjana Prabhu, Jong-Ryul Jeong, D. V. Morales

**Affiliations:** 1 Radiation and Materials Physics Lab, Department of Studies in Physics, Davanagere University, Shivagangotri, Davanagere, 577007 Karnataka India; 2 Nano and Functional Materials (NFML) Lab, Department of Physics, Manipal Institute of Technology, Manipal Academy of Higher Education, Manipal, 576104 Karnataka India; 3 Laboratorio de Nanocompuestos, Departamento de Ingeniería de Materiales (DIMAT), Facultad de Ingeniería (FI), Universidad de Concepción (UdeC), Concepción, Chile; 4 Laboratorio de Nanociencias y Nanotecnología, Facultad de Ciencias Físico Matemáticas (FCFM), Universidad Autónoma de Nuevo León (UANL), 66451 San Nicolás de los Garza, Nuevo León Mexico; 5 Department of Chemistry, M.S. Ramaiah University of Applied Sciences, Bangalore, Karnataka India; 6 Department of Materials Science and Engineering, Graduate School of Energy Science and Technology, Chungnam National University, Daejeon, 34134 South Korea; 7 Department of Environmental Chemistry, Faculty of Sciences, Centro de Energía; and Centro de Investigación en Biodiversidad y Ambientes Sustentables (CIBAS), Universidad Católica de la Santísima Concepción (UCSC), Concepción, Chile

**Keywords:** Gamma irradiation, FDTD modeling, Thermal conductivity, Effusivity

## Abstract

In this article, we present a straightforward in-situ approach for producing Ag NPs incorporated in graphene oxide (GO) blended with glutaraldehyde (GA) cross-linked polyvinyl alcohol (PVA) matrix. Samples are γ-irradiated by doses of 2, 5, and 10 kGy and in comparison with the pristine films, the thermal conductivity (‘*k*’) and effusivity are measured. ‘*k*’ decreases with irradiation doses up to 5 kGy and further increase in the dosage results increase in ‘*k*’. We performed FDTD modeling to verify the effect of polarization and periodicity on the absorptivity and emissivity spectra that are correlated to the ‘*k*’ and effusivity, empirically. Hence, we can confess that the structural properties of the prepared hybrid nanocomposite are manipulated by γ-irradiation. This attests that the PVA/GO-Ag/GA nanocomposite is radiation-sensitive and could be employed for thermal management systems. Moreover, their strong electrical insulation, as the measured dc conductivity of the γ-irradiated samples is found to be in the range of 2.66 × 10^−8^–4.319 × 10^−7^ Sm^−1^, which is below the percolation threshold of 1.0 × 10^−6^ Sm^−1^, demonstrates that they are excellent candidates for the use of thermal management materials. The low ‘*k*’ values allow us to use this promising material as thermal insulating substrates in microsensors and microsystems. They are also great choices for usage as wire and cable insulation in nuclear reactors due to their superior electrical insulation.

**Graphical Abstract:**

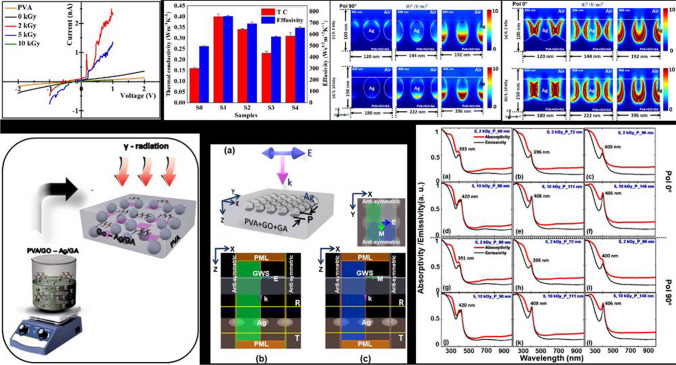

**Supplementary Information:**

The online version contains supplementary material available at 10.1186/s11671-024-03972-3.

## Introduction

Thermal interface materials (TIMs) based on polymer dielectrics encounter low thermal conductivity (*k*) and can be modified by adding high k fillers [1]. Attaining high ‘*k*’ under a lower volume fraction while leveraging the thermal resistance at the interface between polymer matrix and filler [2] is a prodigious task. However, 2D nanosheets can be good fillers prevailing unique surficial properties, but result in dampened ‘*k*’ [3]. Phonon spectrum mismatch between polymer matrix and 2D nanosheets is responsible for this diminishment. Thus, regulating the ‘*k*’ path through filler-filler configuration can transcend the negative effect of interfacial thermal resistance [4] between filler and polymer matrix [5]. Thermal management materials (TMMs) with superior ‘*k*’ are very much necessary to exploit heat dissipation in modern applications for off-peak electricity storage, electronic circuits, energy-saving buildings, chemical reactors, data center industries, and thermal resonators [6–9]. Hence, blending 2D nanosheets with polymeric nanocomposites for designing a flexible, cost-effective, and lightweight filler-filler matrix of high ‘*k*’ can allow us to exploit the waste heat for stable and reliable energy harvesting devices [10–12].

Thermal effusivity or inertia delineates the thermal energy-transferring ability of a material, which is the direct square root product of ‘*k*’ and heat capacity [13, 14]. It is salient that the precise control of effusivity can directly manifest the ‘*k*’. Nano-enhanced phase change materials (NEPCMs) can drastically enhance the thermophysical properties by means of ‘*k*’, such as phase transition temperature, rheological effects, and chemical stability [15, 16]. Besides hybridization with nanoparticles (NPs) and hydrated salts can synergistically yield high thermal storage materials to achieve superior performance of devices [17, 18]. Among the numerous materials, silver (Ag NPs) possess exceptional thermal and electrical properties [16, 19–22] having the capability of augmenting static and transient thermal gradients [23, 24], which pre-eminently function with high ‘*k*’ fluctuations. The thermal gradients (∇T) have an intriguing influence on energy harvesting techniques such as photothermal (photovoltaic), mechanical (electromagnetic, electrostatic, piezoelectric), thermal (temperature gradient, temperature variation in pyroelectrics) [25, 26]. Photothermal responses based on Ag NPs demonstrate exceptional bonding and antibonding plasmonic resonance localization in optical frequencies, which can be meticulously tuned by manipulating the structural, morphological, and dispersion of NPs in these polymeric nanocomposites. Nonetheless, the periodicity (P) of NP dimers (having close proximity) driven plasmon hybridization will unveil resonances that govern the amount of photothermal heat generated owing to their plasmonic bonding modes. The localized surface plasmon resonance (LSPR) modes at resonant frequency (ω_LSPR_) result in scattering and absorption, which predominantly depend on the structural and chemical properties of the NPs, based polymer composites. Henceforth, conscientious and diligent maneuvering of these structural and chemical properties will fetch controllable absorptivity that can ultimately steer the thermal effusivity and ‘*k*’, accordingly.

That being the case, enhancing the structural and chemical properties of the 2D nanosheet blended polymer nanocomposites is a pioneering work in achieving enhanced photothermally induced heat energy production. Despite the biodegradable related disadvantages, their essential usage is accounted for their superior ‘*k*’ in radiation-sensitive and radiation-resistant applications [27, 28]. In this study, we prepared Ag NPs incorporated (in-situ) in graphene oxide (GO) blended with glutaraldehyde (GA) cross-linked polyvinyl alcohol (PVA) matrix in in-situ, simply referred to as “PVA/GO-Ag/GA”. Due to their numerous potential applications in the fields of energy storage and conversion, biosensors, catalysts, photocatalysts, surface-enhanced Raman scattering (SERS), diagnostics, imaging, drug delivery, and antibacterial agents, metal-decorated graphene oxide composites have attracted a lot of interest in the scientific community [29, 30]. Thanks to the thermal characteristics of graphene and its compounds [31, 32], the GO can act as a great filler facilitating heterogeneous nucleation sites to promote polymer crystallization and the heterogeneous nucleation effect is more effective at low filler concentrations [33]. γ-irradiation of these hybrid polymer nanocomposites is a proven non-destructive industrial technique that is widely employed to revamp their structural properties. Long polymer chains are either cross-linked and/or scissile when subjected to high-energy radiations like γ-rays [34]. Both scissile or cross-linking are typically favored by each other. In the pursuit of effective nanoscale device fabrication, harnessing substantial incident light becomes pivotal, leveraging the characteristics of NPs such as size, shape, orientation, and polarization, along with the properties of the surrounding medium. An overarching objective of our study is to fine-tune thermal conductivity by strategically manipulating the structural, morphological, and dispersion aspects of NPs in polymeric nanocomposites through gamma irradiation at relatively low doses. This deliberate control over structural and chemical properties opens avenues for achieving adjustable thermal conductivity, offering potential applications in thermal management systems. Subsequently, we can regulate the photothermal-driven effusivity and ‘*k*’, respectively, which have fundamental impacts on heat insulation efficiency.

On the other hand, the ‘*k*’ that is dependent on optical absorptivity owing to the presence of NPs surrounded by polymer matrix, holds a straightforward relation with emissivity [35]. Gustav Kirchhoff's law of thermal radiation states that given an object in thermal equilibrium with the surrounding radiation field consists of identical absorptivity and emissivity for every frequency, direction, and polarization [36]. It connotes, the more an object can absorb electromagnetic (EM) radiation, the more it can also emit the same kind of radiation ensuring less reflection and scattering. Compositional nanospheres like heteromolecular trimers (Au–Ag–Au) display twofold absorption and heat generation in a finite and isolated subwavelength structure [37]. To perceive this, we have carried out a finite difference time domain (FDTD) numerical investigation on the PVA/GO-Ag/GA matrix, evaluating the absorptivity and emissivity spectra under P-polarized (0°) and S-polarized (90°) radiation. It is evident that the polarization has negligible influence on the observed absorptivity and emissivity spectra for all periodicities at the nanoscale regime ascribing to the structural symmetry of NPs. This acquaints us with the validation of Kirchhoff's law; however, the light-matter interaction is unalike for both polarization states of the incident radiation. This is attested by the photothermal-induced power absorptivity profiles, which unveil the enhanced heat energy harvesting capability of PVA/GO-Ag/GA that can be correlated to thermal effusivity and ‘*k*’. Nevertheless, the thermal effusivity and ‘*k*’ plots are leveraging with the electrical characterization of γ-irradiated (of doses 0, 2, 5, 10 kGy) PVA/GO-Ag/GA matrix also authenticate the aforementioned results confessing that the proposed work can unveil heat conservation mechanism for fabricating the next generation TIMs.

## Experimental section

### Materials and methods

We purchased the silver nitrate (AgNO_3_), PVA (MW about 1, 15,000), sulphuric acid (H_2_SO_4_), and graphite powder (98% purity) from Loba Chemie Private Limited. The high-grade chemicals hydrogen peroxide (H_2_O_2_), sodium nitrate (NaNO_3_), potassium permanganate (KMNO_4_), and hydrochloric acid (HCl) were supplied by Merck Life Science Pvt. Ltd. glutaraldehyde was purchased from SD Fine Chem. Limited.

### The synthesis of the GO

GO was prepared using Hummer's method [38]. Here, 50 ml of sulphuric acid and 3 g of graphite powder were combined and stirred constantly in an ice bath. The mixture was then mixed with 9 g of potassium permanganate and 3 g of sodium nitrate. The liquid was constantly kept stirring at room temperature after being removed from the ice bath. 5 ml of deionized water was added after stirring the mixture for 15 min at a steady temperature of 90 °C. To get rid of any potassium permanganate and manganese dioxide that might still be present, 150 ml of deionized water and 150 ml of hydrogen peroxide were added to the heated mixture. Then the pH was neutralized by washing after collecting the GO slurry.

### Fabricating the PVA/GO-Ag/GA films

20 ml of deionized water with 0.5 weight percent of GO was sonicated for an hour. Later, 20 ml of H_2_O was added to PVA along with constant stirring and heating, until the mixture turned clear aqueous solution. Sonicated GO solution was then added to this and the mixture was stirred for an hour at 50 °C. The solution was constantly kept at a temperature of 50 °C for 1 more hour while being stirred and added 0.1 weight percent AgNO_3_ and 2 ml (1 vol%) of GA. Further, the pristine films (0 kGy) were γ-irradiated by 2 kGy, 5 kGy, and 10 kGy doses and the samples were denoted as shown in Table A1 (supplementary information).

### Irradiation studies

The gamma irradiation process was conducted at the Centre for Application of Radioisotopes and Radiation Technology (CARRT), Mangalore University, Mangalagangotri, Konaje—574,199, Karnataka, India. The specifications of the gamma irradiation chamber 5000 include a Co_60_ source capacity (maximum) of 13,455 Ci (497.8 TBq at the time of irradiation). The dose rate at maximum capacity is 9.5 kGy/hr, with radial dose rate uniformity of + 25% or better.

The irradiation chamber has a sample chamber volume of 5 L and dimensions of 17.2 cm (diameter) × 20.5 cm (height). The chamber is constructed with lead and stainless steel for effective shielding. The overall unit size is 125 cm (length) × 106.5 cm (width) × 150 cm (height), and the irradiation time range starts from 6 s onwards.

### Characterization

Infrared Fourier Transform (FTIR) spectra were collected using a Bruker OPUS 7.0 Alpha spectrometer, covering the range of 400–4000 cm^−1^. These spectra allowed us to investigate interfacial interactions involving PVA. UV–vis transmittance spectra of polymer nanocomposites were obtained utilizing a UV–vis spectrometer with a 200–800 nm wavelength range and was specifically a Shimadzu UV-1800 spectrophotometer. The X-ray diffractometer used for recording the XRD patterns of the samples was the Rigaku Ultima IV, Using Cu K radiation (wavelength: 0.1541 nm). The thermal conductivity measurements were conducted using the Modified Transient Plane Source (MTPS) method, incorporating C-Therm's single-sided sensor with guard ring technology. This sensor is adept at accommodating a wide array of sample types, ranging from solids and liquids to powders and pastes. Offering a measurement range of 0–500 W/mK and a temperature range of − 50 to 500 °C, the MTPS sensor ensures comprehensive thermal conductivity characterization. To guarantee accuracy, samples were meticulously prepared with a flat edge, ensuring a minimum diameter of 18 mm for optimal contact with the sensor. This methodology ensures the reliability and precision of the acquired thermal conductivity data.

## Results and discussions

### Morphological analysis

**Fig. 1:**
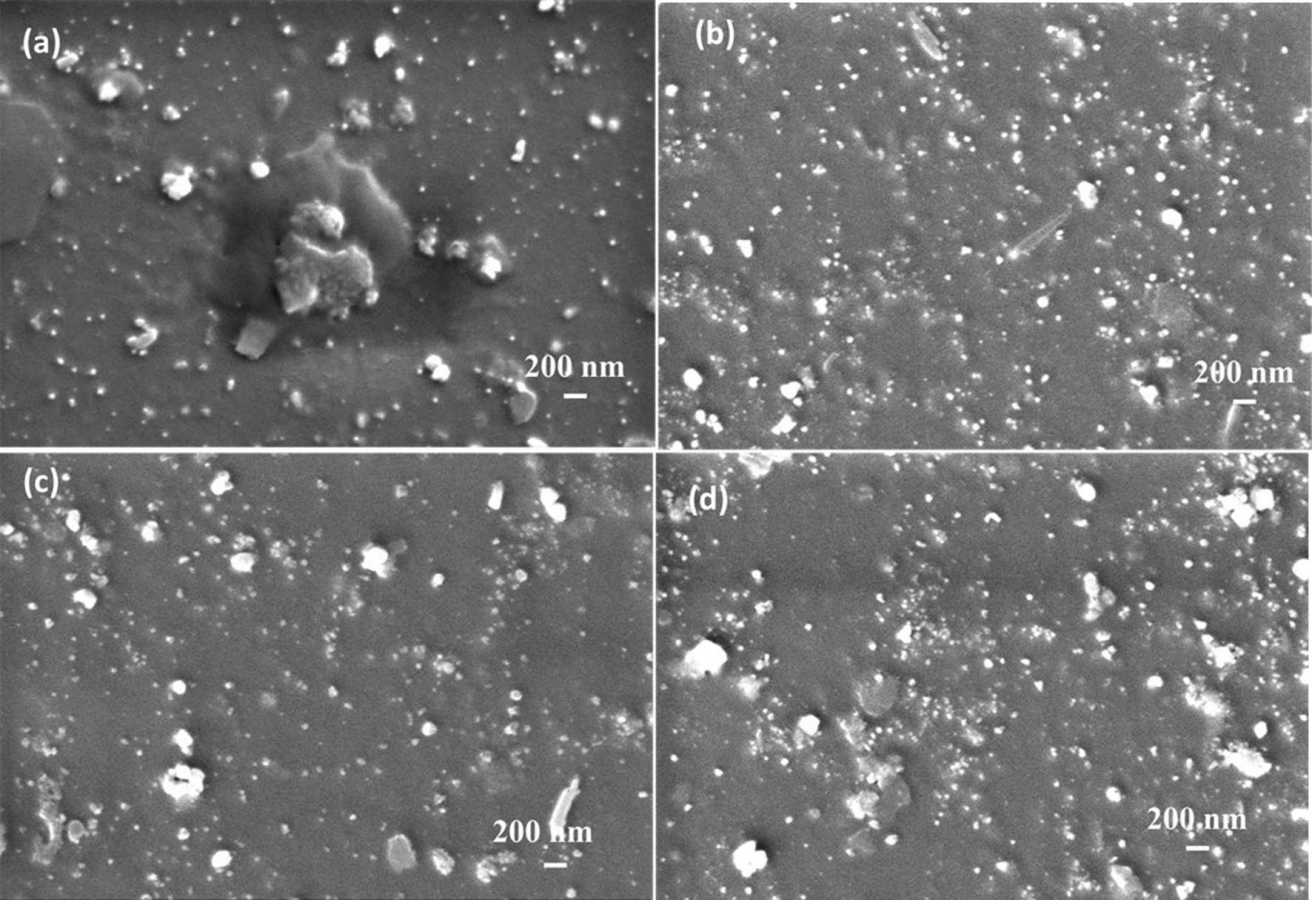
SEM images of **a** pristine PVA/GO-Ag/GA, and γ-irradiated PVA/GO-Ag/GA **b** 2 kGy, **c** 5 kGy, and **d** 10 kGy, correspondingly

Figure 1 represents the surface morphology of PVA/GO-Ag/GA films with and without γ-irradiation. Figure 1a is 0 kGy film (S1) while Fig. 1b–d refers to the 2, 5, and 10 kGy γ-irradiated films (S2, S3, and, S4), respectively. We can see in Fig. 1a the distributed layers of GO in the PVA matrix after the addition of fillers to pristine PVA (Fig. A2) (supplementary information) as a result of GA crosslinking GO and the PVA matrix. Additionally, white bright spots that appear on the PVA crosslinked GO matrix show that Ag NPs have formed, and the size of the Ag NPs is shown in the supplementary Fig. A1a (supplementary information). As a result of γ-irradiation, the surface texture of the nanocomposite has sleeked. Nevertheless, the size of Ag NPs increases upon γ-irradiation as seen in Fig. A1a–c (supplementary information), but for the dose of 10 kGy, the size is decreased (Fig. A1d) (supplementary information). This is ascribed to the saturation of the influence of γ-irradiation occurring after 5 kGy, which caused the internal structural changes justified by the FTIR analysis described in Fig. A3a (supplementary information) in the supplementary information. This is elucidated by observing the γ-irradiated PVA/GO-Ag/GA nanocomposite film's spectra in comparison to the unirradiated film, which caused the polymer's crystalline structure to deteriorate as explained in Fig. A3b (supplementary information). The XRD patterns of unirradiated and γ-irradiated PVA and PVA/GO-Ag/GA are given in Fig. A3b (supplementary information) and crystallite data are summarized in Table A2 (supplementary information).

### Photothermal studies

**Fig. 2:**
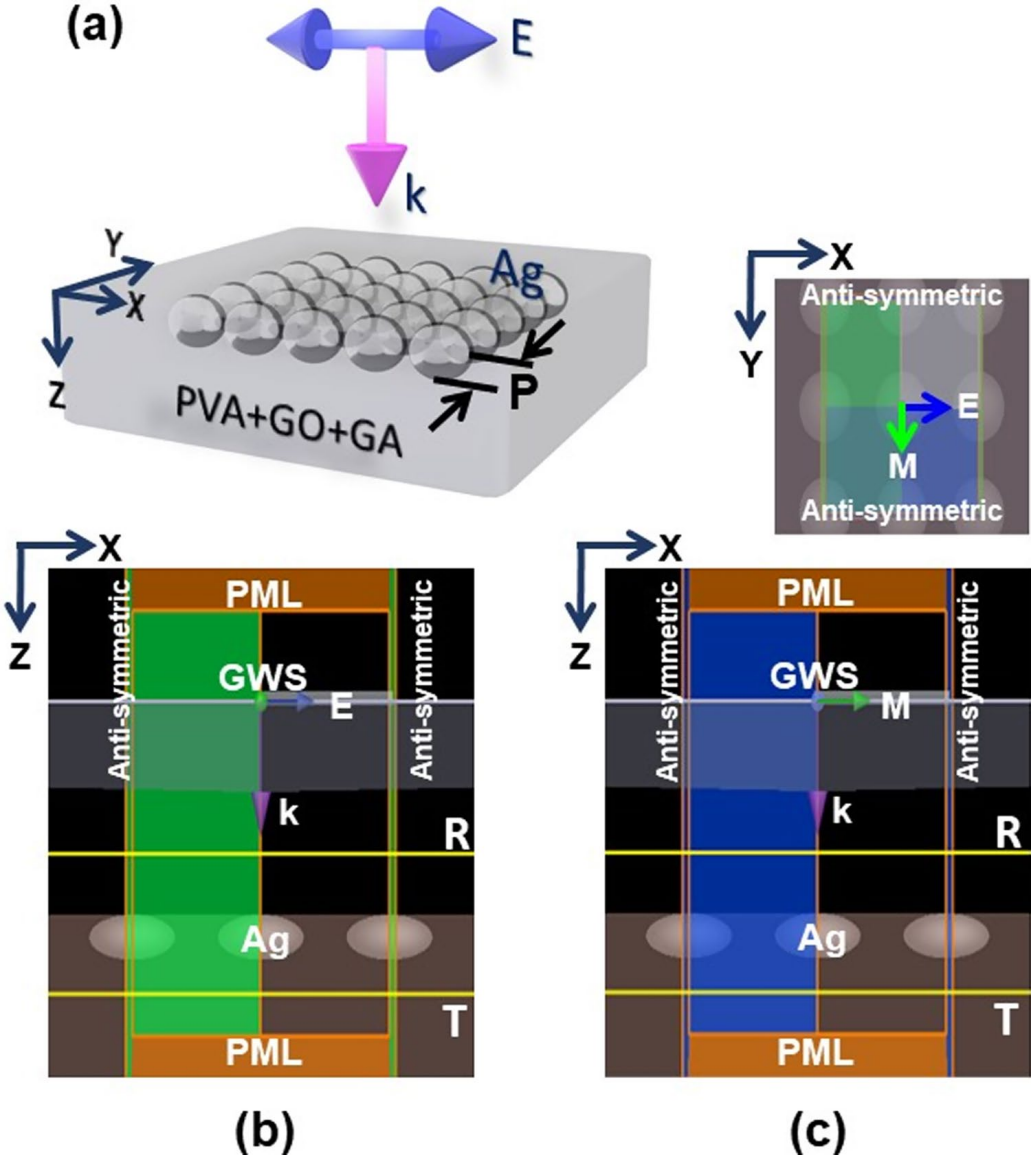
FDTD simulation modeling of PVA/GO-Ag/ GA of **a** schematic representation of the structure illuminated by the Gaussian wave source in the Z-direction (here, P is the periodicity of Ag NPs, E-electric, M-magnetic field vectors, k-propagation vector, and R, T are transmission monitors to capture the power transmitted) **b** polarization 0° (or P-polarization), **c** polarization 90° (or S-polarization) under symmetry boundary conditions (BCs), correspondingly. The XY plane representation from the top view is shown for the E-, M-field vector orientation with reference to the BCs

To discern the optothermal absorptivity and emissivity in the presence of Ag NPs surrounded by polymer matrix in relation to emissivity in a finite and isolated subwavelength structure (i.e. PVA/GO-Ag/GA), we performed an FDTD modeling at 300 K as illustrated in Fig. 2. With reference to Fig. A1 (supplementary information), the average diameter of Ag NPs is calculated as 48, 50, 82, and 72 nm for the γ-irradiation doses 0, 2, 5, and 10 kGy, respectively. Complementing the closely matching diameters of NPs of 0, 2 kGy, and 5, 10 kGy, accordingly, we considered the average diameter (d) of 48, 74 nm in the simulation to investigate the absorptivity and emissivity under the influence of polarization irrespective of the geometrical symmetry. This is to analyze the thermal absorptivity effect in terms of the structural changes resulting in terms of particle size and periodicity. Therefore, we illuminated the structure as shown in Fig. 2a with a Gaussian wave source (GWS) whose electric field vector is oscillating along the X-axis while propagating in the Z-direction. The Ag NPs [with optical constants of [39]] are submerged in the PVA/GO/GA polymer matrix where the ratio of optical constants of PVA: GO: GA is approximated based on the experimental molar ratios as 96:3:1 [40–42]. Figure 2b, c are the P-, S-polarization states of EM waves irradiating the structure under symmetry boundary conditions (BCs). The difference is that the electric field vector should always oscillate parallel to the anti-symmetric BCs owing to the laws of EM radiation. This is explicated with the top (XY) plane view of the structure above Fig. 2c as a reference. Perfectly matched layer BCs are chosen in the GWS direction to clear the dissipation of normally incident radiation at the boundaries in order to avoid reflections from walls.

**Fig. 3:**
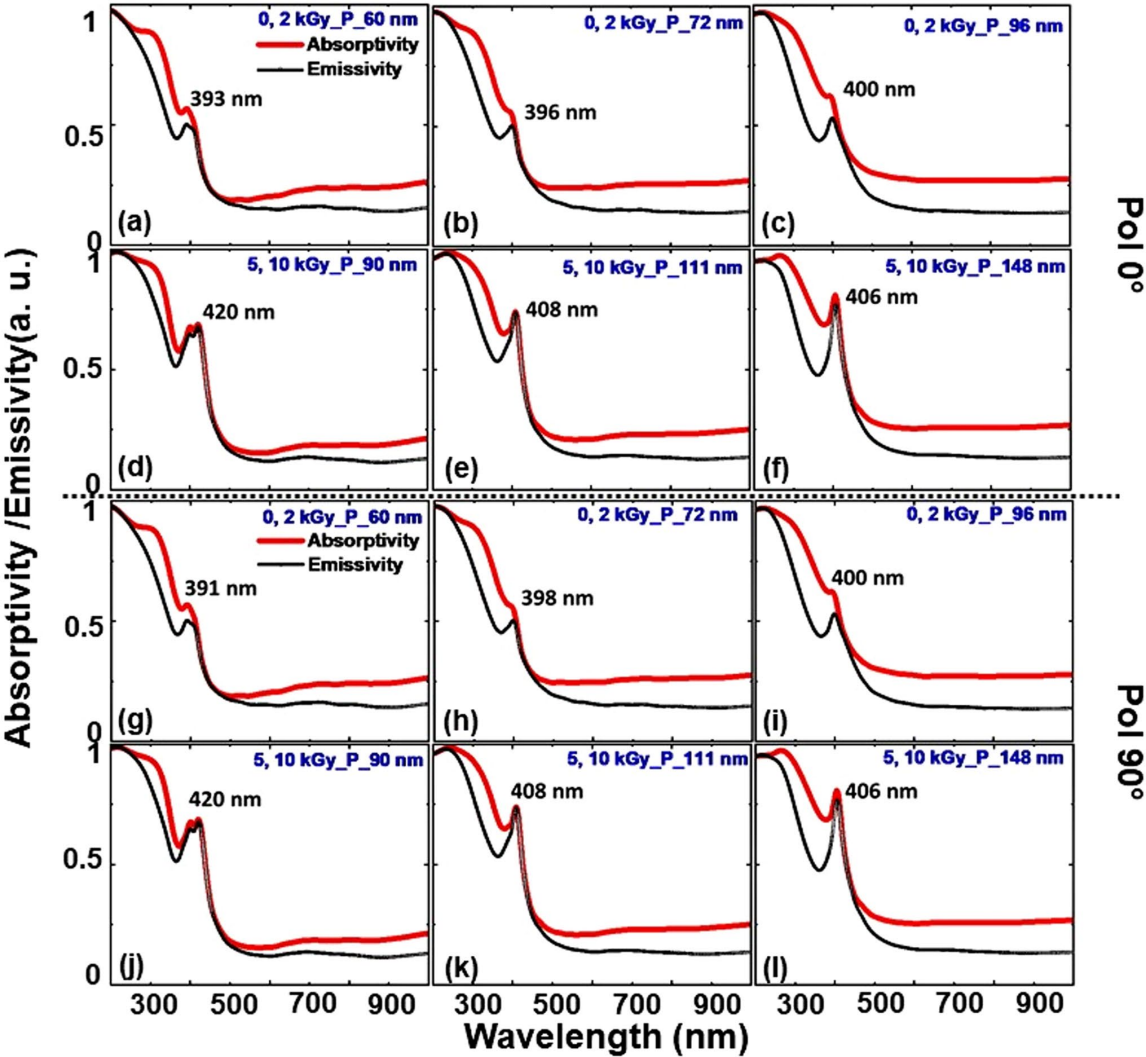
**a**–**f** P-Polarization and **g**–**l** S-polarization dependent absorptivity and emissivity spectra with LSPR resonance wavelength for the 0, 2, 5, and 10 kGy based NP sizes. **a**–**c**, **g**–**i** correspond to the periodicity 60, 72, 96 nm, and **d–f, j–l** correspond to the periodicity 90, 111, and 148 nm with respect to the polarization state of incident radiation, correspondingly

Figure 3 explains the polarization-dependent absorptivity and emissivity spectra of Ag NPs dispersed in the PVA/GO/GA polymer matrix. Both curves are in good agreement with a slight discrepancy, as the power absorbed is a bit higher than emitted. This is because the material can never be a perfect blackbody to emit an equal amount of power absorbed when interaction with EM radiation takes place. The diameter of the Ag NPs is taken as 48 nm (corresponding to 0, 2 kGy) and 74 nm (corresponding to 5, 10 kGy) for this study with reference to Fig. A1 (supplementary information). The periodicity of NPs is intentionally varied as 60, 72, and 96 nm for 48 nm, and 90, 111, and 148 nm for 74 nm, to examine the influence of power absorptivity by NPs with respect to the close proximity to each other in response to the incoming radiation.

As we observe, the LSPR absorptivity peak of Ag is left shifted more or less around 400 nm except for the P = 90 nm for the d = 74 nm, in both polarization states of GWS. When the NPs are in close proximity, the LSPR wavelength tends to approach lower wavelengths below 400 nm (P = 60, 72, 96 nm) for both polarization states. The shift in absorptivity is accounted for by the opaque nature of metals below the plasmon frequency i.e. 420 nm for Ag NPs. However, for the higher diameters (74 nm) the left shift is observed but starting from 420 nm. In terms of absorption, the incident light, being normal, can couple into outgoing radiation modes (in transmission or reflection) that conserve the wave vector up to a reciprocal lattice vector in a direction of discrete periodicity.

**Fig. 4:**
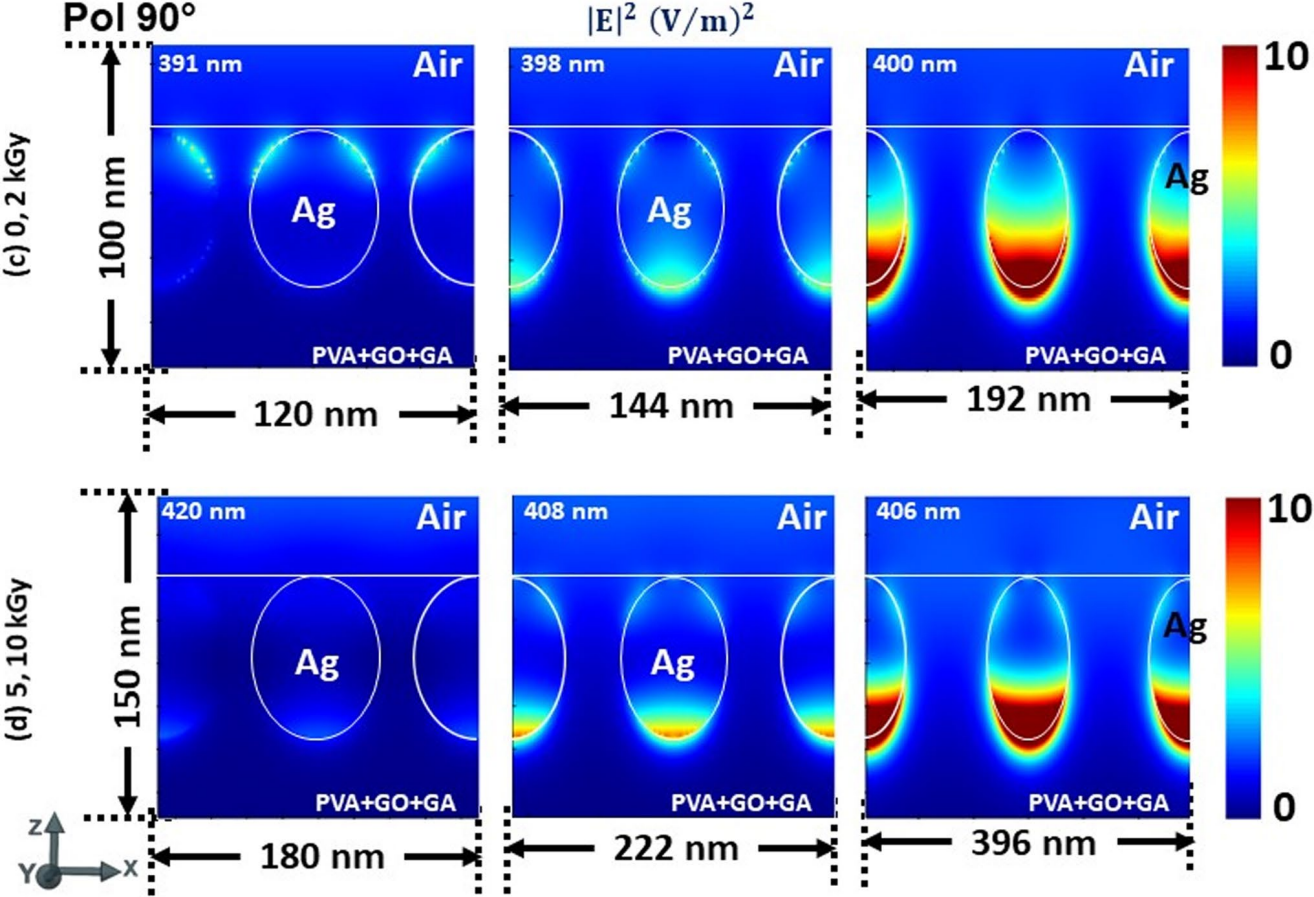
Electric field intensity profiles at the respective wavelengths referred to in Fig. 3 for both P-, and S-polarization states of normally illuminated PVA/GO-Ag/GA structure, respectively

To confess, the electric field intensity profiles are captured in Fig. 4a, b for both P-, 4(c, d) for S-polarization states at the respective wavelengths obtained for the corresponding ‘P’ and ‘d’ values referring to the 0, 2, 5, 10 kGy radiation doses in Fig. 3, accordingly. For better illustration, we have normalized the scale of the profiles to 0–10. Although there is no variation in the LSPR wavelength of absorption for respective periodicities in Fig. 3, we can witness that the structure is disparate in response to the incoming radiation. The electric field intensity distribution across the surfaces is shared among the surfaces of NPs when they are in close proximity and solely confining on the surfaces while they move distance from each other. Surface plasmons (SPs) are excitations that exist on the interface between a plane metal and a dielectric. They are confined to the surface but can propagate freely within that surface. This is similar for both P-, and S-polarization states irrespective of size and periodicity. However, for the S-polarization (90°) state it is trivial to evidence at lower periodicities with a 0–10 scale as given in Fig. 4c, d. To verify this, we have obtained the profiles at a lower scale, which are given in Fig. A3 (supplementary information). For the d = 48 nm (P = 60 nm; at 391 nm), we can see that the field is localized at the interface of the top surface of NP and dielectric-air interface when the NPs are in close proximity. Whereas, at 398 nm (P = 72 nm) the field is more dominant at the bottom surface while at 400 nm (P = 96 nm) it is concentrated only at the bottom, correspondingly. In the case of d = 74 nm, the LSPR wavelength 420 nm (P = 90 nm) shows the field is intending to localize at the bottom. But the 408 nm (P = 111 nm), both top and bottom surfaces try to confine the field while it is dominant at the bottom. For the 406 nm (P = 148 nm) it is only confined at the bottom of the NP. This can be interpreted as the fabricated structure being polarization sensitive and enabling it as an efficient and potential material for photothermovoltaic applications.

**Fig. 5:**
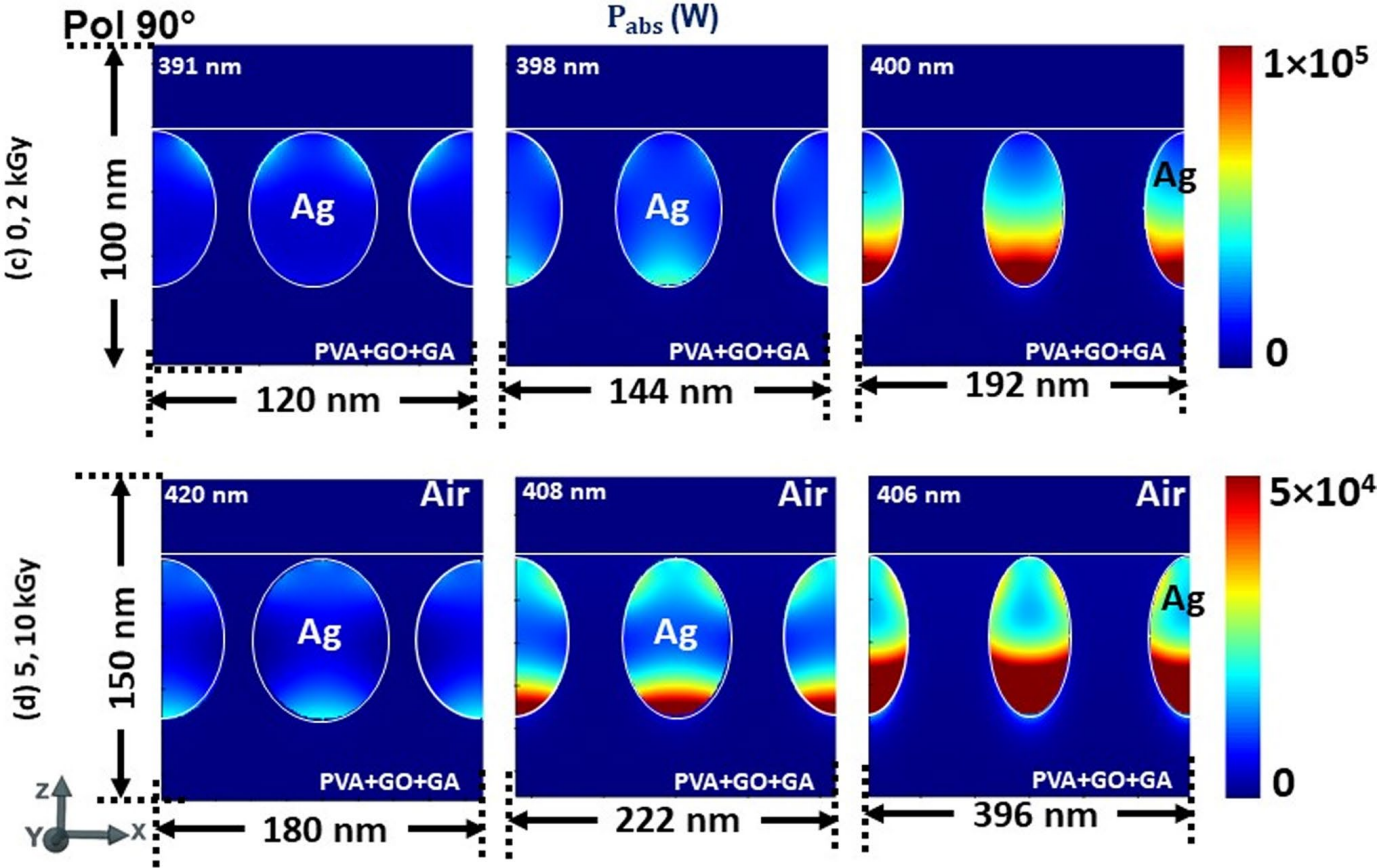
Power absorbed in normally illuminated PVA/GO-Ag/GA structure at the respective wavelengths referred to in Fig. 3 for both P-, and S-polarization states, respectively

To justify, we have captured the power absorption profiles and is summarized in Fig. 5 for the corresponding LSPR wavelengths mentioned for the d, P values in Fig. 3. Likewise, for the electric field intensity distribution for the polarization 90°, we have plotted the profiles at a lower scale to elucidate the power absorption spots within the NPs in Fig. A4 (supplementary information). This attests to our observation in Fig. 4 and also validates that the thermal absorptivity or emissivity is sensitive to the polarization and periodicity of NPs surrounded by the dielectric polymer matrix under thermal equilibrium.

### Thermal conductivity

Correlating the thermal absorptivity profiles obtained from the modeling we have investigated the thermal conductivity of the prepared PVA/GO-Ag/GA hybrid nanocomposite. The main heat carriers in metals are typically electrons, whereas the main heat carriers in polymers are phonons. In reality, it is conceivable that in the ordered structure of crystals, when an atom in the lattice vibrates, it transfers vibrational energy to the surrounding atoms, which in turn starts to oscillate and spread the energy further into the sample. Thermally, amorphous materials can be viewed as a collection of many imperfections that prevent heat transport. Although atoms can vibrate slightly close to the equilibrium position in the ordered molecular chains of crystalline regions of polymers, where heat transfers quickly, persistent random entanglement causes phonon scattering and affects phonon transport, leading to low k values [1, 43].

Using the following Eq. (1), the thermal conductivity (k) of nanocomposites was determined: ρ is the density of the nanocomposites, C_p_ is the specific heat, and σ is the thermal diffusivity, resulting in

1 $$k=\rho {C}_{p}\sigma$$

**Fig. 6:**
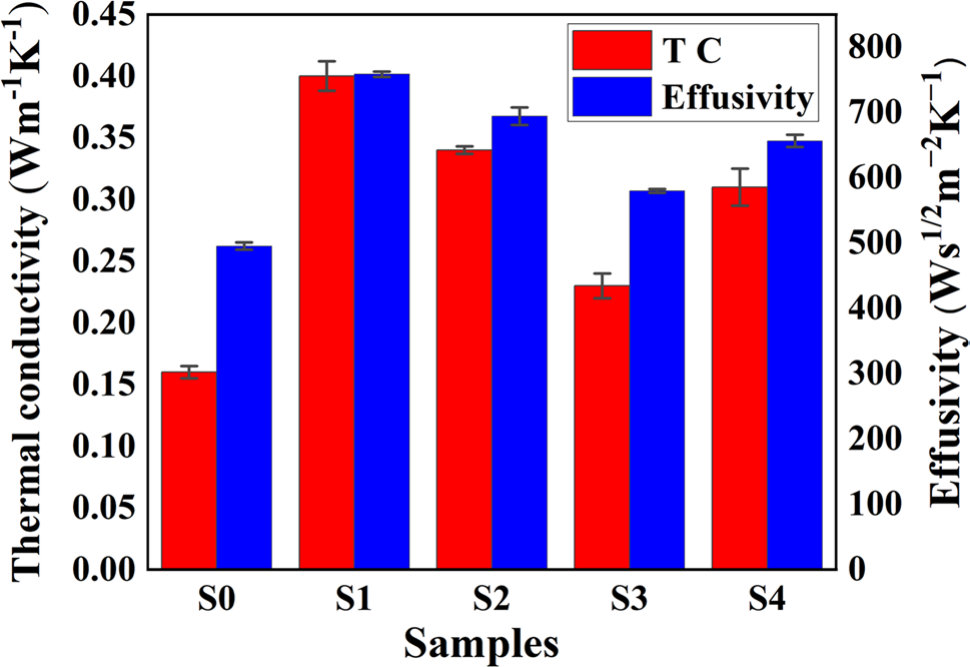
Thermal conductivity and effusivity comparative plot of pristine PVA and gamma irradiated PVA/GO-Ag/GA

Figure 6 depicts the thermal conductivity of nanocomposites irradiated for different gamma dosages. In the case of S1, the addition of nanocomposites boosts the thermal conductivity of PVA (Table 1), by effectively bridging the neighboring PVA-Ag-GA layers with GO, Ag NPs assist in maintaining the continuity of the thermal conductive network and create efficient thermally conductive routes, providing PVA composites with better thermal functionality. Reduced effective phonon scattering centers resulted in decreased interfacial thermal resistance due to the synergistic action of GO and Ag and improved heat conductivity [10].

**Table 1:** Thermal conductivity, thermal conductivity enhancement, and effusivity of the irradiated and unirradiated samples

Samples	k (Wm^−1^ K^−1^)	$TCE= ({k}_{n}-{k}_{p})/{k}_{p}$	Effusivity (Ws^1/2^ m^−2^ K^−1^)
S0	0.158 ± 0.01	–	495 ± 5.732
S1	0.40 ± 0.01	1.53	758.4 ± 3.98
S2	0.34 ± 0.01	1.15	694.2 ± 13.47
S3	0.23 ± 0.01	0.46	579.8 ± 2.82
S4	0.31 ± 0.01	0.96	656.4 ± 9.26

As seen in Fig. 6, when the nanocomposite is subjected to radiation, the thermal conductivity drops because energy deposits may cause ionization, excitation, and the breaking of chemical bonds, which will lead to the formation of numerous free radical species. These radiation-induced free radicals instantaneously interact with the oxygen dissolved in the polymer nanocomposite to cause phonon scattering [31, 32]. Also according to a study by Lorenzo Mirizzi et al., amorphous materials have low thermal conductivity (k), due to the lack of crystalline structures. Defects (such as voids, entanglements, chain ends, and impurities) give rise to excess scattering events and reduce thermal conductivity [44] and Table A2 (supplementary information) shows that calculated values of crystallinity index decrease for S2 indicating the amorphous nature of the sample. As a result, the 2 kGy dose of radiation reduces the thermal conductivity of nanocomposites, k value decreases for S3. For sample S4, the k value increases. This is due to the fact that, as shown in Table A2 (supplementary information), an increase in irradiation dose causes the composites' crystallinity to increase, which raises the k value.

When comparing the thermal conductivity of nanocomposites with the pure matrix, thermal conductivity enhancement (TCE) was taken into account and given by [17]

2 $$TCE=({k}_{n}-{k}_{p})/{k}_{p}$$

where kp and kn stand for PVA’s and nanocomposites’ respective thermal conductivities. When compared to pure PVA, the TCE of the unirradiated sample was found to be 1.53 as noticed in Table 1, which is higher than irradiated nanocomposites.

### Thermal effusivity

In addition to thermal conductivity, which is an important thermal property for heat conduction in solids, thermal effusivity (e) is a highly significant thermal parameter that controls the processes of heat transmission and heat exchange for solids and liquids. The relationship between these quantities is provided by [45]

3 $$e=\sqrt{k\rho c}$$

where c is the specific heat capacity, ρ is the mass density and *k* is the thermal conductivity Ws^1/2^ m^−2^ K^−1^. The obtained thermal effusivity values for S1 show an improvement with the addition of filler content as seen in Fig. 6 and Table 1, demonstrating better heat exchange and transfer than S0. This is because when crystallinity rises, effective thermal pathways are created. For samples S2 and S3, irradiation causes a decrease in crystallinity, which leads to a decreasing phonon mean free path that causes excessive phonon scattering and hence lowers effusivity. Additionally, as the dose is increased, the crystallinity rises, favoring the improvement of effusivity [46].

### Electrical conductivity

**Fig. 7:**
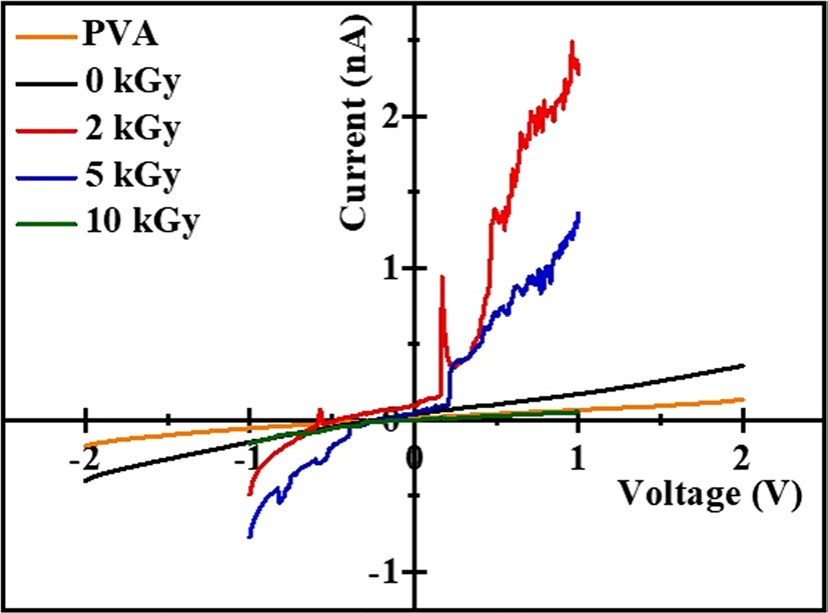
Current versus Voltage characteristics of PVA/GO-Ag/GA films under the 0, 2, 5, and 10 kGy irradiation doses

I-V (current–voltage) characteristics of the PVA/GO-Ag/GA have been carried out in the voltage range of -2 to 2 V in light of the fact that the electrical insulation of the filler likely had an impact on the use of nanocomposites in electronic devices. The plot of I-V characteristics of irradiated and unirradiated PVA/GO-Ag/GA nanocomposites is presented in Fig. 7. The measured values of electrical resistance and conductivity are depicted in Table 2. It is found that the dc (direct current) conductivity of S0 is 6.72 × 10^−9^ Sm^−1^, when nanofillers are added to GA cross-linked PVA, conductivity was found to have increased by one order (2.32 × 10^−8^ Sm^−1^). The rise in dc conductivity is caused by the creation of a charge transfer complex between the nanofillers and the cross-linked PVA matrix. Ag creates charge transfer complexes, lowering the barrier between the conducting channel and the trapping site, causing conduction at grain boundaries.

**Table 2:** Resistance and electrical conductivity of irradiated and unirradiated samples

Samples	Resistance (Ω)	${\sigma }_{dc}= t/RA S{m}^{-1}$
S0	1.638 × 10^10^	6.72 × 10^–9^
S1	4.73 × 10^9^	2.32 × 10^–8^
S2	2.55 × 10^8^	4.319 × 10^–7^
S3	5.88 × 10^8^	1.87 × 10^–7^
S4	4.14 × 10^9^	2.66 × 10^–8^

The secondary electrons produced during ionization undergo collision with other electrons in the nanocomposite, resulting in energy deposition and localized heating. This localized heating due above processes may introduce structural changes that adversely affect the electrical conductivity of the nanocomposite. When nanocomposites are exposed to γ-radiation dc conductivity increases and is found to be 4.319 × 10^−7^ Sm^−1^ for S2, this is because of the production of ions and free radicals, initially, some of them are trapped in the sample as the dose increases trapped charges are released and allows the easy movement of the charge carrier which increase the orderliness of dipole groups [47]. As the gamma dose increased to 5 kGy and 10 kGy conductivity decreased to 1.87 × 10^−7^ Sm^−1^ and 2.66 × 10^**−8**^ Sm^−1^ respectively, this is because charges are tightly packed in the matrix due to an increase in crystallinity. However, in comparison with the electrical conductivity of graphene-based polymer nanocomposite (1.0 × 10^–6^ Sm^−1^) at the percolation threshold [17], dc conductivity values of the PVA/GO-Ag/GA samples are far below. This indicates that the presence of insulating GO preventing the formation of electrical transfer pathways.

Generally, materials with enhanced electrical conductivity also exhibit higher thermal conductivity, especially if the conductive pathways for charge carriers coincide with paths for heat conduction. The relationship between electrical and thermal conductivity can be complex and is influenced by factors such as the type of fillers, their distribution, and the overall composite structure.

In our composite, after the sample is irradiated to 2 kGy electrical conductivity rises from 2.32 × 10^−8^ to 4.319 × 10^−7^ Sm^−1^. This might be due to the fact that as seen in Fig. A3a (supplementary information), the OH peak of S2 broadens after irradiation, indicating a decrease in the OH group due to bond breaking and chain scission. Additionally, a decline in the intensity of bands at 1421 and 1373 cm^−1^ suggests the decoupling of Ag–O and Ag-C bonds due to weak interactions between C-H and O–H vibrations. These structural changes, induced by irradiation, result in defects, the formation of free radicals, and a reduction in crystallinity, ultimately leading to increased electrical conductivity. Whereas thermal conductivity decreases from 0.40 ± 0.01 to 0.34 ± 0.01 Wm^−1^ K^−1^. The weak interaction between contacted fillers can contribute to high contact thermal resistance, leading to poor thermal coupling between the filler and matrix. This typically results in limited enhancement of thermal conductance in the composite due to a significant mismatch in the phonon spectra of the interface components.

Table 2 shows that electrical conductivity decreases with an increase in dose from 5 kGy onwards. This is because crystallinity reverts as PVA macromolecules crosslink, enhancing molecular mass. This crosslinking surrounds the nanoparticle, reducing nanoparticle mobility and, consequently, decreasing electrical conductivity. As the dose increases, the molecular mass around the nanoparticle further increases, leading to a continuous decrease in electrical conductivity. Contrastingly Table 1 reveals that thermal conductivity decreases up to 5 kGy and further increases for 10 kGy owing to an increase in crystallinity as evident from XRD. This divergence is because thermal properties exhibit marked differences from electrical properties in composites. The electrical properties of the organic matrix are negligible, with only the conductive fillers contributing to electrical conductivity. In contrast, for thermal transport, there is no absolute thermal insulator, and the contribution from thermal transport, through the organic matrix cannot be ignored. In summary, the observed changes in thermal and electrical properties at different irradiation doses are intricately linked to mechanisms such as secondary electron collisions, energy transfer to phonons, and radiolytic heating, highlighting the complex interplay between gamma radiation and the PVA/GO-Ag/Glutaraldehyde nanocomposite.

To the best of our knowledge, there have been very limited studies on the effect of gamma irradiation on the thermal conductivity of polymer nanocomposites. Table 3 shows that a recent study by Tarawneh MA et al. focused on the radiation effect on the thermal conductivity of TPE (thermoplastic elastomer) /CNT (carbon nanotube)/nanoclay, revealing an increase in conductivity with a dose increase from 0 to 150 kGy. However, with a further increase in the dose to 200 to 250 kGy, the thermal conductivity decreases to that of the unirradiated state.

**Table 3:** Comparison of the effect of gamma irradiation on thermal conductivity of PVA/Ag-GO/GA and TPE/CNT/nanoclay nanocomposites

Doses kGy	Samples	k (Wm^−1^ K^−1^)	References
0	S1	0.40 ± 0.01	This work
	TPE/CNT/nanoclay	0.124	[27]
2	S2	0.34 ± 0.01	This work
5	S3	0.23 ± 0.01	
10	S4	0.31 ± 0.01	
100	TPE/CNT/nanoclay	0.153	[27]
150	TPE/CNT/nanoclay	0.164	
200	TPE/CNT/nanoclay	0.143	
250	TPE/CNT/nanoclay	0.112	

In our work, the thermal conductivity value is found to be 0.4 Wm^−1^ K^−1^ for 0 kGy, and a decreasing trend is observed from 2 to 5 kGy doses. Interestingly, with an increase in the dose to 10 kGy, the thermal conductivity value shows an increase. This consistent change in thermal conductivity values indicates that the material can be tuned for the required thermal conductivity by gamma irradiation in thermal management systems. The merit of our work lies in tailoring the thermal conductivity at a relatively low gamma dose.

## Conclusions

In this paper, we have prepared Ag NPs incorporated in graphene oxide (GO) blended with glutaraldehyde (GA) cross-linked polyvinyl alcohol (PVA) matrix by in-situ method, simply referred to as “PVA/GO-Ag/GA”. Upon γ-irradiating these films with the 0, 2, 5, and 10 kGy doses in order to structurally modify them for the enhancement of electrical and thermal properties. Using the FDTD modeling, we have studied the optothermal properties that are correlated with the thermal conductivity and effusivity of the hybrid nanocomposite. The prepared structure exhibits excellent thermal stability with respect to the polarization of incident radiation and makes it a very good candidate for photothermovoltaic applications. The results of the measurements show that at room temperature, thermal conductivity (k) initially falls with irradiation doses and increases as the dosage increases. With increasing dose, dc electrical conductivity first rises and then falls. An electrical conductivity of 4.319 × 10^−7^ Sm^−1^ was observed for the sample exposed to a 2 kGy dose, which is below the value of 1.0 × 10^–6^ Sm^−1^ observed at the percolation threshold. Studies on thermal and electrical performance show that these composite materials have outstanding suitability for the application of thermal management materials. These materials are found to be promising materials as thermal insulating substrates in microsensors and microsystems owing to the low thermal conductivity values. Along with that, their strong electrical insulation demonstrates that they are excellent candidates for use as wire and cable insulation in nuclear reactors.

### Supplementary information: This information contains

Comparison of particle size of pristine (a) 0 kGy and γ-irradiated PVA/GO-Ag/ GA (b) 2 kGy, (c) 5 kGy, and (d) 10 kGy, correspondingly.
SEM image of pristine PVA
a)FTIR spectra and (b) XRD patterns of pristine (0 kGy) and γ-irradiated PVA/GO-Ag/ GA nanocomposite films [48–52]
Electric field intensity profiles at the respective wavelengths referred to in Fig. 3 for S-polarization states of normally illuminated PVA/GO-Ag/GA structure, respectively.
Power absorbed in normally illuminated PVA/GO-Ag/GA structure at the respective wavelengths referred to in Fig. 3 for S-polarization states, respectively.
Representation of samples with different irradiation doses.
The crystallinity index and crystallite size values of PVA/GO-Ag/GA were obtained from XRD data.

### Supplementary Information

Additional file1 (DOCX 1198 KB)

## Data Availability

The authors declare that the data supporting the findings of this study are available within the paper and its supplementary information files.
