# Supplementary material for: Modification of thermal and electrical characteristics of hybrid polymer nanocomposites through gamma irradiation for advanced applications
Source: Discov Nano. 2024 Feb 22;19(1):34. doi: 10.1186/s11671-024-03972-3 (PMC10884377; doi:10.1186/s11671-024-03972-3)
Supplement: Supplementary file 1 — Additional file1 (DOCX 1198 KB) [file 11671_2024_3972_MOESM1_ESM.docx]

**Supplementary Information**

**Modification of thermal and electrical characteristics of hybrid polymer nanocomposites through gamma irradiation for advanced applications**

Kavitha C M^1^, Eshwarappa K M^1^*, Shivakumar Jagadish Shetty^2^, Gurumurthy S C^2*^, Srivathsava Surabhi^3, 4^, Niranjana Prabhu T^5^, Jong-Ryul Jeong^6^, D. V. Morales^7^

*^1^Radiation and Materials Physics Lab, Department of Studies in Physics, Davanagere University, Shivagangotri, Davanagere - 577007, Karnataka, India.*

*^2^Nano and Functional Materials (NFML) Lab, Department of Physics, Manipal Institute of Technology, Manipal Academy of Higher Education, Manipal - 576104, Karnataka, India.*

*^3^Laboratorio de Nanocompuestos, Departamento de Ingeniería de Materiales (DIMAT), Facultad de Ingeniería (FI), Universidad de Concepción (UdeC), Concepción, Chile.*

*^4^Laboratorio de Nanociencias y Nanotecnología, Facultad de Ciencias Físico Matemáticas (FCFM), Universidad Autónoma de Nuevo León (UANL), San Nicolás de los Garza, Nuevo León, Código Postal 66451, Mexico.*

*^5^Department of Chemistry, M.S. Ramaiah University of Applied Sciences, Bangalore, Karnataka, India.*

*^6^Department of Materials Science and Engineering, Graduate School of Energy Science and Technology, Chungnam National University, Daejeon- 34134, South Korea.*

*^7^Department of Environmental Chemistry, Faculty of Sciences; Centro de Energía; and Centro de investigación en Biodiversidad y Ambientes Sustentables (CIBAS), Universidad Católica de la Santísima Concepción (UCSC), Concepción, Chile.*

***Corresponding authors**: [km.eshwarappa@gmail.com](mailto:km.eshwarappa@gmail.com) (Eshwarappa), gurmurthy.sc@manipal.edu (Gurumurthy)

**Section-1**

| **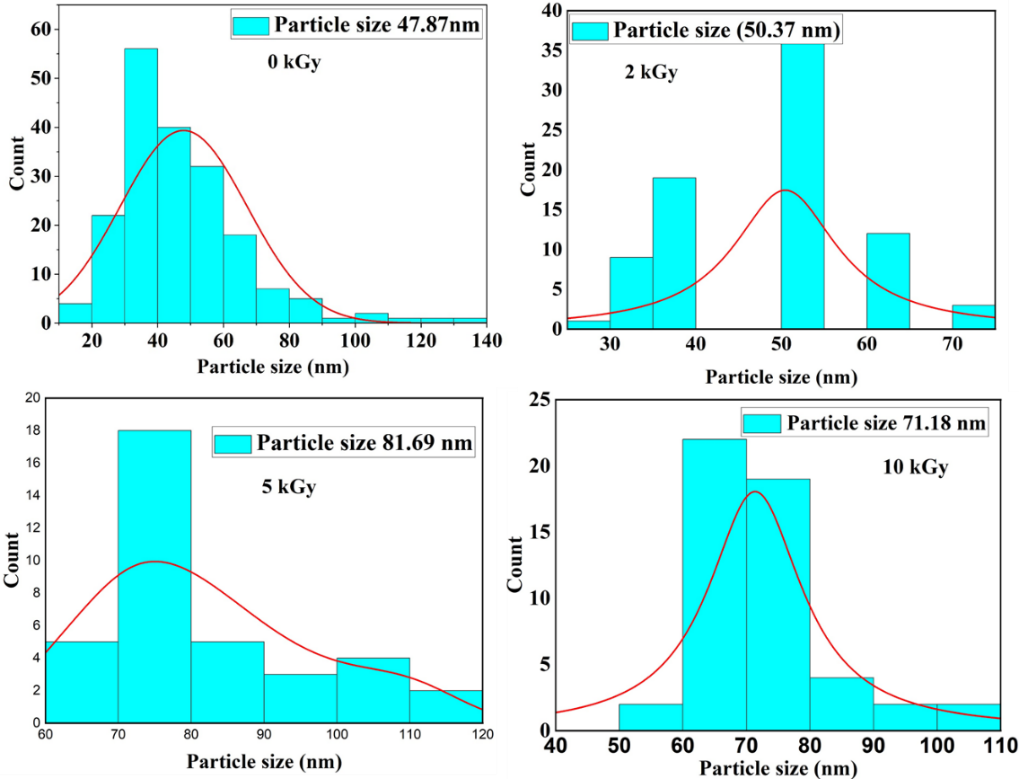** |
| --- |

**Figure A1:** *Comparison of particle size of pristine (a) 0 kGy and* γ-*irradiated PVA/GO-Ag/ GA (b) 2 kGy, (c) 5 kGy, and (d) 10 kGy, correspondingly.*

| *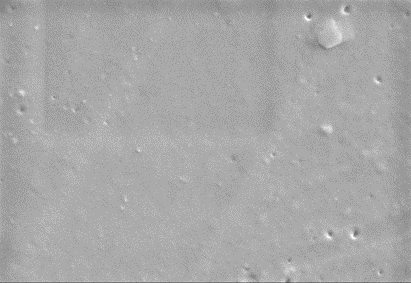* |
| --- |

**Fig. A2:** *SEM image of pristine PVA*

**1μm**

| **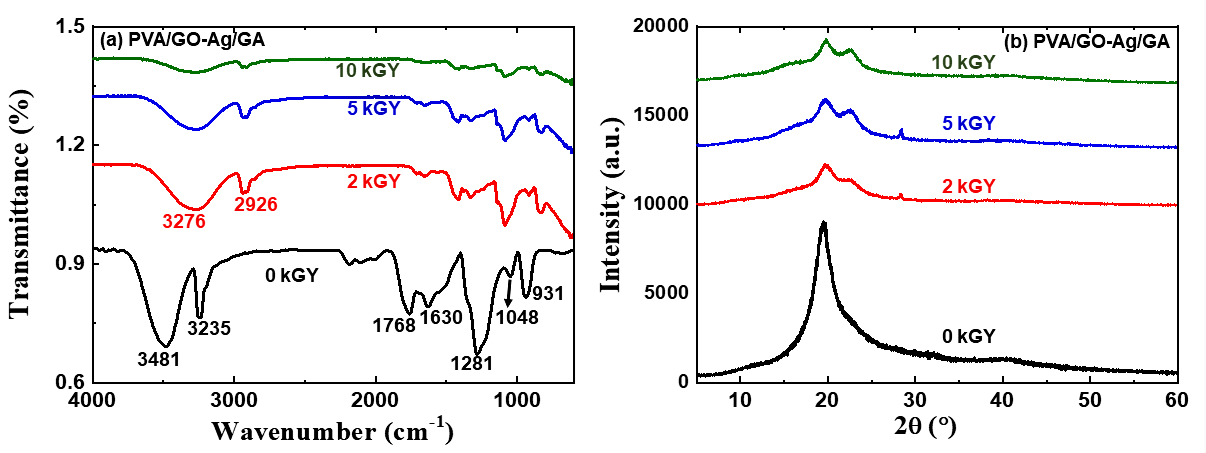** |
| --- |

***Figure A3:*** *(a) FTIR spectra and (b) XRD patterns of pristine (0 kGy) and* γ-*irradiated PVA/GO-Ag/ GA nanocomposite films.*

**FTIR analysis:**

It is clear from Figure A3 (a), in nonirradiated (0 kGy) samples, the OH peak narrows and the band strength rises in comparison to S0 (from our previous publication [39] referred in the main manuscript), indicating an increase in the concentration of the hydroxyl group following crosslinking with GA. There is a significant interaction between PVA and GO, as evidenced by the peak shift in the S1 from 3292 to 3481 cm^-1^. The OH group of PVA and the oxygen group of GO interact with each other during this process to produce hydrogen bonds. High amounts of GO oxidation can strengthen the hydrogen bonds between PVA and GO by increasing the likelihood of forming them. The disappearance of the bands at 1421 and 1373 cm^-1^, which are caused by the interaction of the hydroxyl groups of GA with Ag nanoparticles, provides additional proof of the successful interaction between nanocomposites and a crosslinking agent. This interaction causes the O-H and C-H vibrations to decouple and produce the Ag-O and Ag-C bonds. After γ-irradiation at different dosages, it is observed that the peaks in PVA chains embedded with Ag NPs-decorated on GO move considerably to lower wavenumbers with degraded intensity at 3235 and 1768 cm^-1^. The peaks at the wavenumber in the range of 1200–1500 cm^-1^ practically vanish as the γ-dosage is increased, demonstrating that the PVA chains undergo structural reorganization after γ-irradiation [40] (referred to in the main manuscript).

**XRD analysis:**

The degree of crystallinity was determined by deconvoluting XRD diffractograms using the Gaussian function, and the results are displayed in Table A2 below. The formation of GO. After the addition of fillers in S1 (Ag NPs and GO nanosheets are confirmed by the XRD pattern from our previous publication [39], no peaks matching to fillers were seen, indicating that the GO in the PVA had been totally exfoliated allowing the Ag NPs to be well-dispersed. The interaction between filler and GA cross-linked PVA increases the crystallinity index of PVA, indicating an increase in intermolecular contact between PVA chains. The crystallinity index values are provided in Table A2. Table A2 shows that the size of the crystallites increases when fillers are added. Figure A3 (b) shows that after exposure, the peak intensity of S2 at 2θ = 19.49^°^ decreased, and new peaks emerged at 2θ = 22.47^°^ and 28.36^°^, indicating the deterioration of the films. Additionally, Table A2 demonstrates that low-dose γ-irradiation reduces the crystallinity-producing free radicals. These bond cleavage procedures, such as chain scission or hydrogen elimination, can produce free radicals, which in turn lower the molecular weight of polymers. These free radicals might also be created by cross-linking activities like recombination reactions or other processes like the production of double bonds, which would result in the outermost zone of the crystallite producing a shell of distorted pattern and lowering the actual crystalline volume. Further, it is noted that the crystallinity index of S4 rises as the polymer absorbs and transfers energy from the radiation, which results in bond breaking, ionization, or recombination processes when the γ-irradiation dose is increased. As previously scattered atoms in the outermost crystallites' zones return to their former crystalline locations, the crystallinity index will rise [41–43] (referred to in the main manuscript).

**
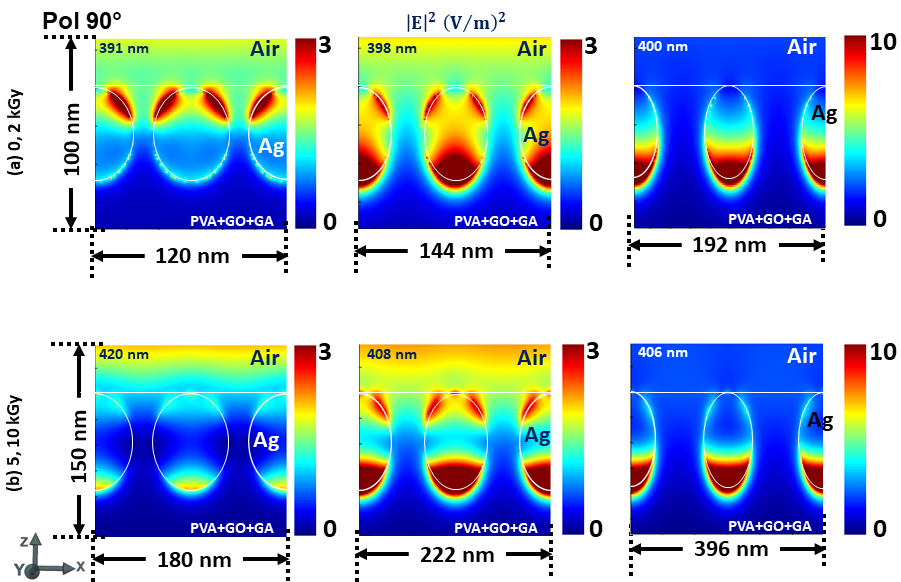
**

***Figure A4:*** *Electric field intensity profiles at the respective wavelengths referred to in Figure 3 for S-polarization states of normally illuminated PVA/GO-Ag/GA structure, respectively.*

**
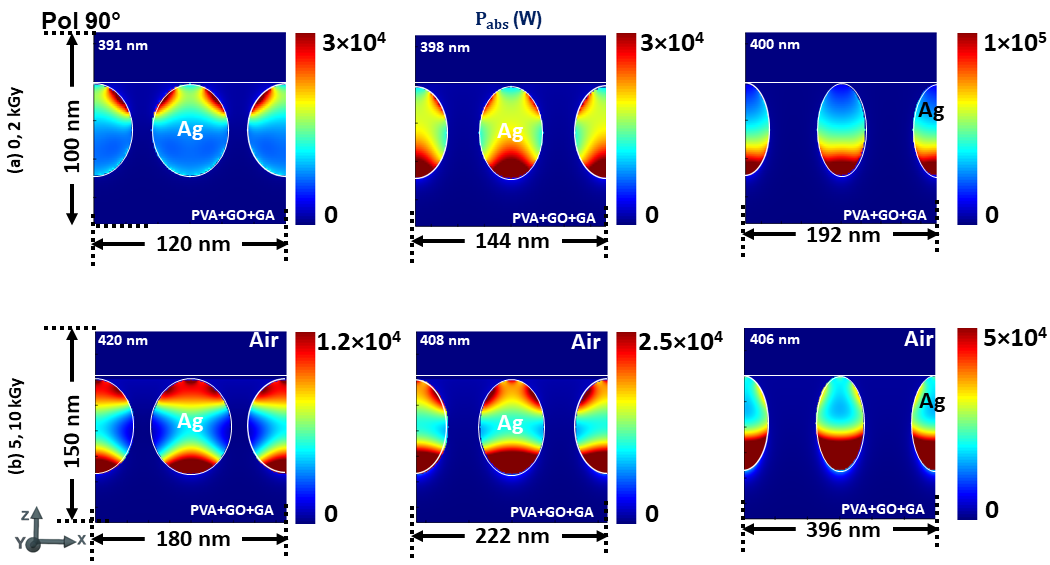
**

**Figure A5:** *Power absorbed in normally illuminated PVA/GO-Ag/GA structure at the respective wavelengths referred to in Figure 3 for S-polarization states, respectively.*

**Section 2:**

***Table A1***: *Representation of samples with different irradiation doses.*

| **Samples with irradiation doses** | **Designation** |
| --- | --- |
| PVA | S0 |
| PVA/GO-Ag/GA 0 kGy | S1 |
| PVA/GO-Ag/GA 2 kGy | S2 |
| PVA/GO-Ag/GA 5 kGy | S3 |
| PVA/GO-Ag/GA 10 kGy | S4 |

**Table A2:** *The crystallinity index and crystallite size values of PVA/GO-Ag/GA obtained from XRD data*.

| **Sample** | **The crystalline peak's total area (x_c_)** | **The peak's total crystalline and amorphous surface area (x_c_+x_a_)** | **Crystallinity index**  $\boldsymbol{X}_{\boldsymbol{c}}\boldsymbol{=}\frac{\boldsymbol{x}_{\boldsymbol{c}}}{\boldsymbol{(}\boldsymbol{x}_{\boldsymbol{c}}\boldsymbol{+}\boldsymbol{x}_{\boldsymbol{a}}\boldsymbol{)}}$ | **Crystallite size (nm)**  $\boldsymbol{D=}\frac{\boldsymbol{K\alpha}}{\boldsymbol{\beta cos\theta}}$ |
| --- | --- | --- | --- | --- |
| **S1** | 15344.40 | 37926.31 | 40.46 | 3.01 |
| **S2** | 12772.04 | 36530.48 | 34.96 | 7.59 |
| **S3** | 17111.2 | 44655.34 | 38.31 | 13.43 |
| **S4** | 6066.82 | 11835.53 | 51.25 | 3.46 |

|  |
| --- |

|  |
| --- |
